# Evaluation of a novel combination of TRAM-34 and ascorbic acid for the treatment of corneal fibrosis *in vivo*

**DOI:** 10.1371/journal.pone.0262046

**Published:** 2022-01-10

**Authors:** Allison A. Fuchs, Praveen K. Balne, Elizabeth A. Giuliano, Nishant R. Sinha, Rajiv R. Mohan

**Affiliations:** 1 Departments of Veterinary Medicine and Surgery and Biomedical Sciences, College of Veterinary Medicine, University of Missouri, Columbia, Missouri, United States of America; 2 Harry S. Truman Memorial Veterans Hospital, Columbia, Missouri, United States of America; 3 Mason Eye Institute, School of Medicine, University of Missouri, Columbia, Missouri, United States of America; Cedars-Sinai Medical Center, UNITED STATES

## Abstract

Corneal injury and aberrant wound healing commonly result in corneal fibrosis and subsequent vision loss. Intermediate-conductance calmodulin/calcium-activated K+ channels (K_Ca_3.1) have been shown to promote fibrosis in non-ocular and ocular tissues via upregulation of transforming growth factor beta (TGFβ). TRAM-34 is a selective inhibitor of K_Ca_3.1 and reduces fibrosis by downregulation of TGFβ-induced transdifferentiation of stromal fibroblasts to myofibroblasts. Ascorbic acid has been demonstrated to be effective in promoting corneal re-epithelialization and reduction of neovascularization via anti-VEGF and anti-MMP mechanisms. This study evaluates tolerability and efficacy of a novel combination of TRAM-34 (25μM) and ascorbic acid (10%) topical treatment for corneal fibrosis using an established *in vivo* rabbit model and conducting clinical eye examinations. Markers of corneal fibrosis were evaluated in all corneas at study endpoint via histopathology, immunofluorescence, and quantitative real-time PCR. The eyedrop treated eyes showed significantly improved clinical outcomes based on modified McDonald Shadduck scores, reduction of clinical haze on Fantes scores, and reduction of central corneal thickness (CCT). At cellular and molecular levels, eyedrop treatment also significantly reduced expression of alpha smooth muscle actin (α-SMA) mRNA and protein, collagen III mRNA, and fibronectin mRNA compared to non-treated eyes. Our study suggests that a tested new bimodal eyedrop is well tolerated and effectively reduces corneal fibrosis/haze in rabbits *in vivo*.

## Introduction

Corneal haze or fibrosis is a common sequela to corneal injury, infection, and ocular surgery in all mammalian species. The long-term sequelae of corneal damage is frequently significant permanent visual loss [1–5]. This can have a major impact on quality of life for both human and veterinary patients.

Corneal wound healing is a complex process involving numerous cytokines, activation of keratocytes, transdifferentiation of fibroblasts to myofibroblasts, angiogenesis, and increased extracellular matrix (ECM) deposition [5–7]. The cytokine transforming growth factor beta (TGFβ) has been shown to play a major role in the formation of corneal fibrosis via activation of fibroblasts and differentiation of activated fibroblasts to myofibroblasts [7–9]. It is well established that the persistence of opaque myofibroblasts in the corneal stroma leads to corneal haze and stromal opacity, and that a reduction in myofibroblasts correlates with improved corneal transparency following corneal injury [5, 7, 10, 11]. A variety of therapeutic strategies including gene therapy, mitomycin C, pirfenidone, vorinostat, and others have demonstrated that reduction of TGFβ will reduce myofibroblast formation, thus inhibiting corneal fibrosis [1, 2, 12–20]. However, some of these therapeutic modalities can have negative side effects and very few are approved for clinical use at present [21].

TRAM-34 (1-[(2-chlorophenyl) diphenylmethyl]-1*H*-pyrazole) is a selective inhibitor of intermediate-conductance calmodulin/calcium-activated K+ channels (K_Ca_3.1) [22]. These channels are expressed in mitochondrial and cytoplasmic membranes and are known to assist with regulation of cell cycle progression and proliferation [23]. K_Ca_3.1 channels are upregulated in response to injury [24–26]. Previous studies have shown activation of K_Ca_3.1 to be important in the development of fibrosis in various organ systems such as the lung, liver, and kidney [26–28]. Recently, the peer-reviewed literature has demonstrated the role of K_Ca_3.1 in corneal cell proliferation and its importance in corneal fibrosis [24]. *In vitro* corneal cell culture experiments have demonstrated that K_Ca_3.1 mediates the TGFβ-1 induced proliferation and differentiation of fibroblasts to myofibroblasts [24, 25]. Inhibition of K_Ca_3.1 by TRAM-34 downregulates these processes and thus may present a therapeutic target for treatment and prevention of corneal fibrosis [29].

Ascorbic acid is abundant in the corneal epithelium of various species and has antioxidant properties as well as protective effects in corneal disease [30]. Application of topical ascorbic acid has been associated with improved corneal epithelial wound healing *in vivo* and is thought to aid in reconstruction of epithelial basement membranes as well as upregulating corneal epithelial stem cell formation [31]. Studies in rabbits *in vivo* have shown reduction of corneal neovascularization with the use of topical ascorbic acid via reduction of vascular endothelial growth factor (VEGF) and matrix metalloproteinase-9 (MMP9) [32].

Both TRAM-34 and ascorbic acid have previously been shown to be safe and well-tolerated when administered to the cornea topically with minimal effects or cellular toxicity [24, 25, 31–34]. In this study, we investigate the hypothesis that a dual formulation of topical TRAM-34 and ascorbic acid will be well-tolerated, safe, and effective at reducing corneal haze after wounding in an *in vivo* model.

## Materials and methods

### Animals

Twelve healthy 2 to 3-month-old female New Zealand white rabbits (Charles River Laboratory Inc., Wilmington, MA) weighing 2.5 to 3 kg were utilized for this study. All studies were performed in accordance with the Association for Research in Vision and Ophthalmology (ARVO) statement for the use of animals in ophthalmic and vision research and were approved by the University of Missouri Institutional Animal Care and Use Committee. Following the 3R rule (reduce, replace, and refine) animal rule to keep the number of animals in experiments as low as possible, both eyes of 12 rabbits were utilized. Twenty-four eyes were divided into 4 groups. Group-1 (left eyes: injury cohort) received alkali (n = 6). Group-2 (right eyes: naïve cohort) received BSS onto the normal eye (n = 6). Group-3 (left eyes: therapy cohort) received alkali injury and eyedrop twice daily for 5 days (n = 6). Group-4 (right eyes; safety cohort) received eyedrop twice daily onto the naïve eye for 5 days (n = 6). All rabbits underwent a complete ophthalmic examination by an American Board of Veterinary Ophthalmology approved senior ophthalmology resident prior to onset of the study, including slit lamp biomicroscopy (SL-15 Kowa Company, Ltd, Tokyo, Japan), indirect ophthalmoscopy (Wireless indirect ophthalmoscope, Keeler Instruments Inc., Broomall, PA, USA and pan retinal 2.2 indirect lens, Volk Optical Inc., Mentor, OH, USA). All rabbits were determined to be free of ocular disease.

### *In vivo* corneal wound model

Using an established corneal wound model, corneal alkali wounding was induced in the left eye of each rabbit [35]. Briefly, after initial clinical examinations and extraocular imaging, rabbits were anesthetized by intramuscular injection of ketamine hydrochloride 50mg/kg (MWI, Boise, ID) and xylazine hydrochloride 10mg/kg (Akorn, Lake Forest IL). Proparacaine hydrochloride 0.5% (Alcon, Fort Worth, TX) was topically applied to the cornea and a wire eyelid speculum was placed. A 7mm-diameter filter paper was soaked in 0.5N sodium hydroxide (NaOH) solution and then applied onto the axial cornea for 30 seconds while visualized under a surgical microscope (Leica Wild Microscope MEL53; Leica, Wetzlar, Germany). Following removal of the filter paper, the wounded cornea was immediately and copiously rinsed with sterile balanced salt solution (BSS) to remove residual alkali solution. Fluorescein stain (Flu-Glo, Akorn, Inc., Buffalo Grove, IL, USA) was applied to verify corneal burns.

### Tram-34/ascorbic acid preparation and treatment

The bimodal eyedrop formulation was prepared by solubilizing TRAM-34 (25μM) and ascorbic acid (10%) in balanced salt solution (BSS) and adjusting the pH using either hydrochloric acid (HCl) or sodium hydroxide (NaOH) to achieve a pH approaching 6.4 as assessed by an electronic pH meter at room temperature under sterile conditions.

### Corneal health and corneal haze analysis

Corneal health was evaluated prior to study initiation and at regular intervals throughout the study period. Using slit-lamp biomicroscopy, ocular health was graded according to the modified McDonald-Shadduck (mMs) scoring system [36] and corneas were imaged using a slit-lamp biomicroscope fitted with a digital imaging system (Kowa, portable Vk-2 Version 5.5) as previously described, as well as a stereomicroscope (Leica MZ16F, Leica Microsystems Inc., Buffalo Grove, IL) equipped with a digital camera (SpotCam RT KE, Diagnostic Instruments Inc., Sterling Heights, MI) [20, 37]. Scoring was performed daily for the first 5 days, then at days 7, 14, and 28. Ophthalmic testing including Schirmer tear testing (Fischer Scientific, Pittsburgh, PA, USA), fluorescein staining, applanation intraocular pressure (IOP) measurements (Tono-Pen AVIA, Reichert Technologies, Depew, NY, USA), pachymetry (Accutome AccuPach VI, Keeler Instruments Inc., Broomall, PA, USA), and extraocular imaging was performed at days 0, 3, 7, 14, and 28.

Corneal haze scoring was performed according to the established Fantes grading scale [38]. Haze was scored by three independent examiners (AAF, PKB, SK) masked to the treatment group. In summary, grade 0 is a clear cornea; grade 0.5 is considered clear with trace haze on tangential illumination; grade 1 is minimal haze on direct or diffuse illumination; grade 2 is mild haze easily visible on direct focal slit lamp examination; grade 3 is moderate opacity which partially obscures iris detail; grade 4 is severe opacity that completely obscures iris details at the site of corneal injury.

### Intraocular pressure and central corneal thickness measurement

Intraocular pressure and central corneal thickness (CCT) recordings were measured at day 0, 3, 7, 14, and 28 using an applanation tonometer and ultrasonic pachymeter, respectively. All measurements were performed under general anesthesia after application of topical anesthetic with the rabbits in lateral recumbency and the eye being measured facing up. All IOP measurements were performed between 9am and 11am to minimize diurnal variations.

### Euthanasia and tissue collection

Rabbits were humanely euthanized with intravenous pentobarbital 150mg/kg (SomnaSol, Henry Schein Animal Health, Dublin, OH, USA) while under general anesthesia on day 28 post-injury. Corneas were harvested and halved using sharp dissection. One half of the corneal sections were immediately placed in 24x24x5 mm molds (Fischer Scientific, Pittsburgh, PA, USA) containing optical cutting temperature compound (Tissue Plus O.C.T., Fisher HealthCare, Houston, TX, USA) and snap frozen. Frozen tissue blocks were maintained at -80°C until further processing. The remaining half of the corneal section was placed in a pre-labeled cryo-vial and stored immediately at -80°C until further processing for gene expression studies.

### Histopathology and immunofluorescence studies

Serial corneal sections (8 μm) were prepared from cryo-preserved corneal tissues using a cryostat (HM525 NX UV; Microm GmbH, Walldorf, Germany), placed on labeled glass microscope slides (Superfrost Plus; Fisher Scientific, Pittsburgh, PA, USA), and stored at −80°C until staining. Hematoxylin and eosin (H&E) staining was performed as previously described for histopathologic examination [3, 39]. Tissue sections for immunofluorescence studies were immunostained for alpha smooth muscle actin (α-SMA; M0851; Dako, Carpentaria, CA, USA) using established methods to evaluate for the presence of myofibroblasts in tested corneal tissue [40, 41]. In brief, tissue sections were incubated at room temperature for 30 minutes with 2% bovine serum albumin and then probed with mouse monoclonal anti-α-SMA antibody (1:200 dilution), and incubated at room temperature for 60 min, followed by overnight incubation at 4°C. The sections were then incubated with Alexa-Fluor 488 goat anti-mouse IgG secondary antibody (1:1000 dilution, A11001; Invitrogen, Carlsbad, CA, USA) for 1 hour in darkness at room temperature. Antifade Mounting Medium containing DAPI (H1200, Vector Laboratories, Inc. Burlingame, CA, USA) was used to stain the nucleus and mount corneal sections. The stained corneal sections were imaged using a fluorescence microscope (Leica) and the α-SMA positive cells were quantified in six randomly selected, non-overlapping full thickness central corneal columns, extending from anterior to posterior stromal surfaces.

### RNA extraction, cDNA synthesis, and quantitative reverse transcription polymerase chain reaction

Total RNA was extracted from tissues using the RNeasy kit (Qiagen, Valencia, CA), according to the manufacturer’s protocol and stored at -80°C until analysis. The quantitative reverse transcription polymerase chain reaction (qRT-PCR) was performed using One Step Plus Real-Time PCR system (Applied Biosystems, Carlsbad, CA) according to manufacturer’s instructions as previously described [24, 25, 41]. This reaction mixture was run at universal cycle (95°C for 10 min, 40 cycles at 95°C for 15 s, and 60°C for 60 s) following manufacturer’s instructions. GAPDH was used as a housekeeping gene for α-SMA, fibronectin, and collagen-3, with the primer sequences described in Table 1.

**Table 1:** Quantitative reverse transcription PCR primer sequences.

Gene	Primer sequence (5’-3’)		Tm (°C)
**α-SMA**	**Forward**	TGG GTG ACG AAG CAC AGA GC	60
	**Reverse**	CTT CAG GGG CAA CAC GAA GC	60
**Fibronectin**	**Forward**	GCG CCA CCT ACA ACA TCA TA	60
	**Reverse**	CAC TGG CAC GAG AGC TTA AA	60
**Collagen-3**	**Forward**	AGA ACA CGC AAG GCT GTG AGA CTA	60
	**Reverse**	CCA ACG TCC GCA CCA AAT TCT TGA	60
**GAPDH**	**Forward**	GCC TCA AGA TCA TCA GCA ATG CCT	60
	**Reverse**	TGT GGT CAT GAG TCC TTC CAC GAT	60

The fluorescence threshold value (Ct) was calculated to detect signal differences in association with an exponential increase of PCR products in the log linear phase. Relative expression/fold change over the corresponding values for the control was calculated by the 2-ΔΔCt method. Two to three independent experiments were executed, each sample was run in triplicate, and the average fold changes in mRNA levels were calculated.

### Statistical analysis

Results are expressed as mean ± standard error of the mean (SEM). Statistical analysis was performed using a commercially available software (GraphPad Prism 6.0, GraphPad Software, La Jolla, CA, USA). Kolmogorov-Smirnov’s test was used to determine whether data were normally distributed. Data that were not normally distributed were transformed using the natural log function. Unpaired t-test was performed for α-SMA qPCR safety study. A one-way or two-way ANOVA followed by Bonferroni multiple comparisons test was performed for clinical scoring and tissue processing data, respectively. Results were considered significant at *p* ≤ 0.05.

## Results

### *In vivo* safety and toxicity

**Fig 1:**
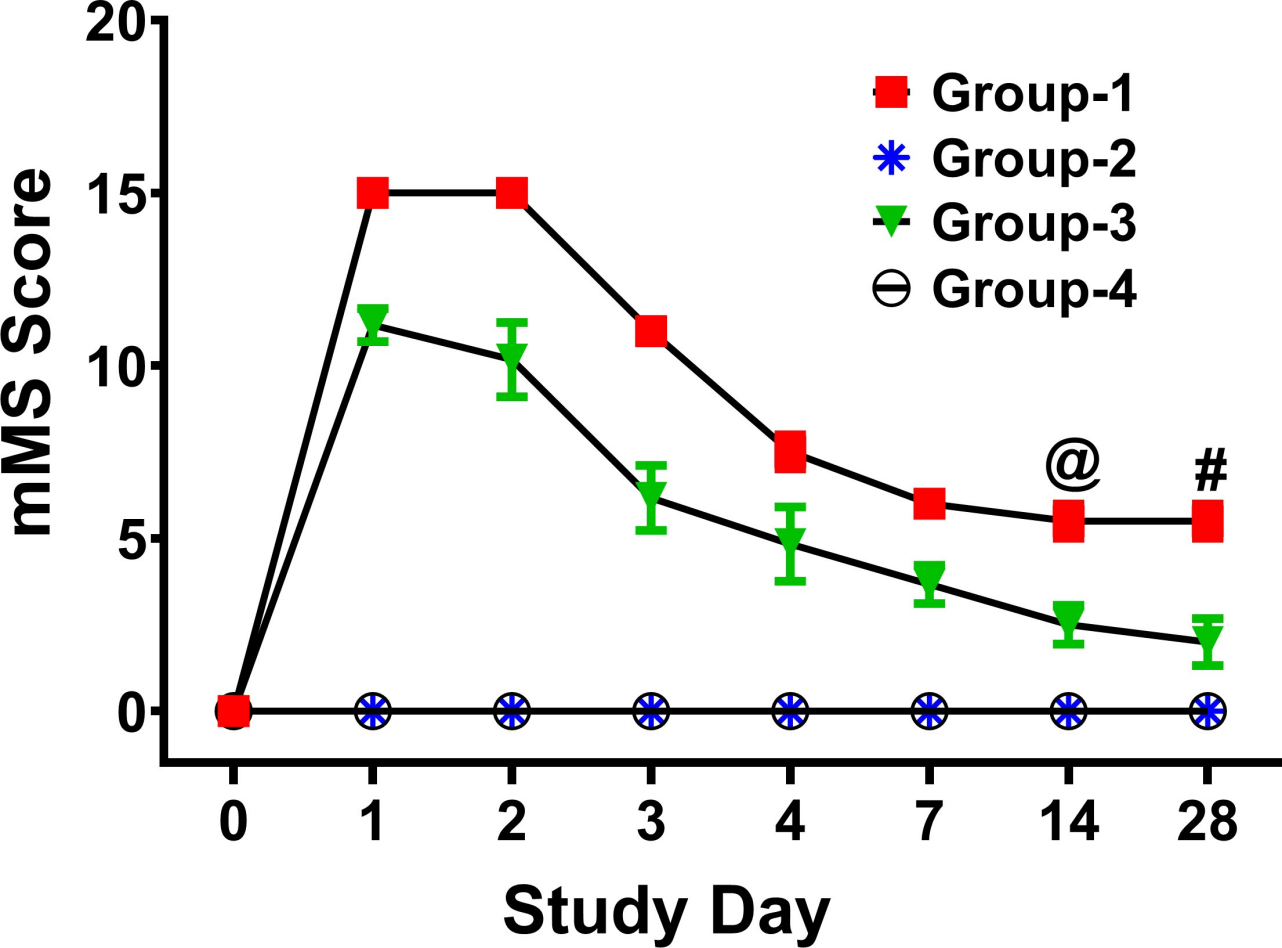
*In vivo* tolerability evaluation with modified McDonald Shadduck scoring (mMS). In safety evaluation, no significant difference in mMS scores between naïve eyes of Group-2 and unwounded eyedrop-treated eyes of Group-4 up to 28 days was observed. Conversely, alkali wounded eyes of Group-1 showed significant difference in mMS compared to the eyes of Group-3 at day-14 and day-28 (n = 6 for each group; @ = *p* value <0.01; # = *p* value <0.001).

**Fig 2:**
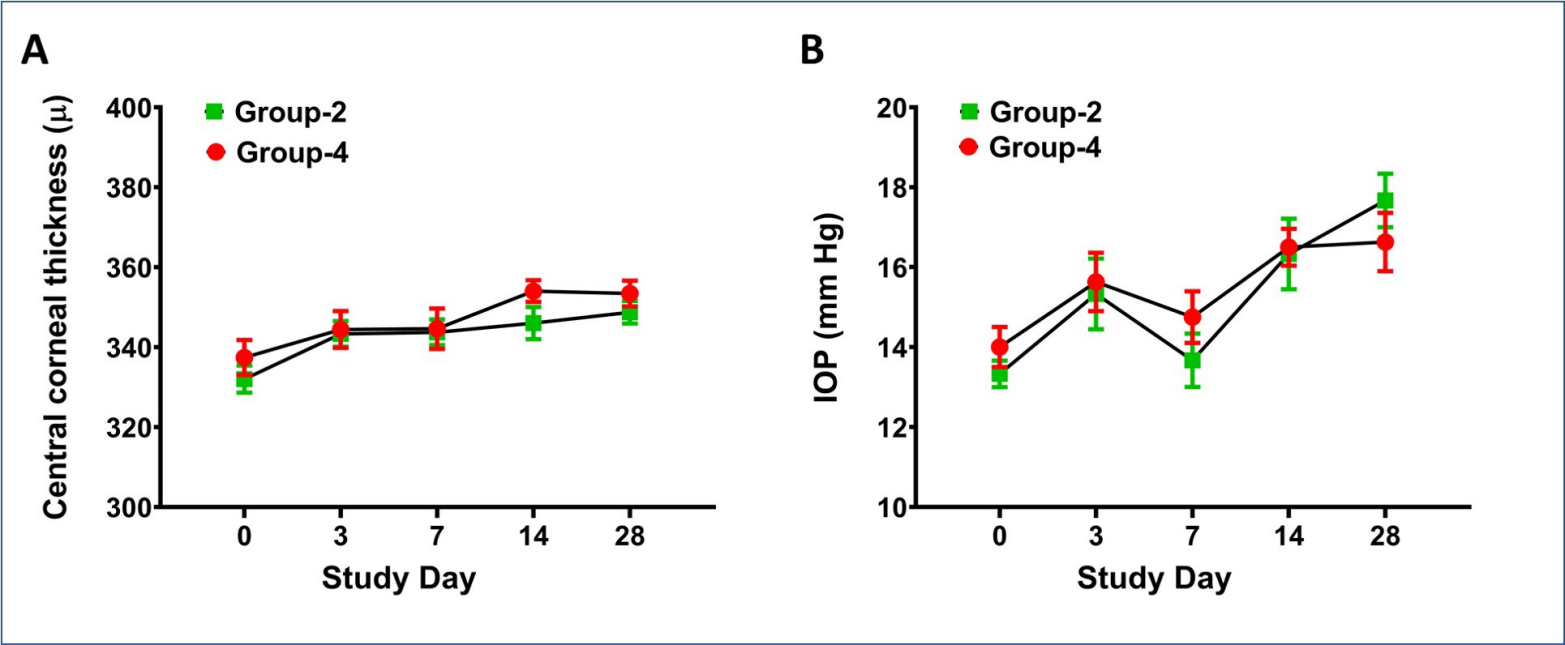
*In vivo* tolerability evaluation showing central corneal thickness and intraocular pressure. No significant difference in central corneal thickness (A) or intraocular pressure (B) in the naïve eyes of Group-2 and unwounded eyedrop-treated eyes of Group-4 was observed until 28 days (n = 6 for each group).

**Fig 3:**
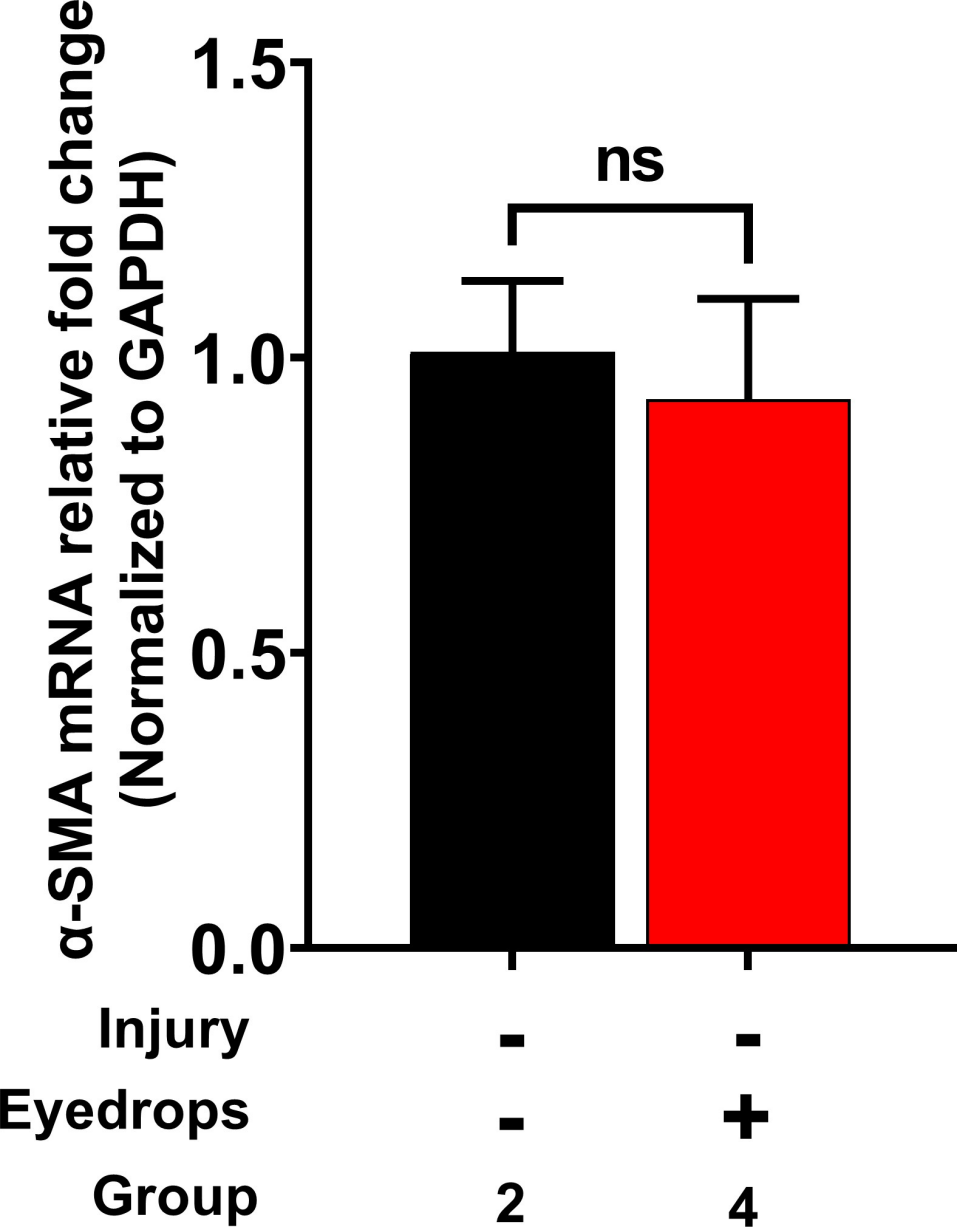
*In vivo* tolerability evaluation showing α-SMA fibrotic expression. The naïve eyes of Group-2 and unwounded eyedrop-treated eyes of Group-4 showed no significant differences in α-SMA gene expression observed until a longest tested time point, 28 days (n = 6 for each group; ns = not significant).

Unwounded rabbit eyes of the Group-2 (naïve cohort) and Group-4 (safety cohort) had mMs scores of zero throughout the study, indicating no ocular irritation (Fig 1). Additionally, Group-2 (naïve cohort) and Group-4 (safety cohort) eyes showed no significant differences in the central corneal thickness (CCT) (Fig 2A) or IOP (Fig 2B) as well as any fluorescein uptake (data not shown) at all tested times in the study. Also, molecular analysis of fibrotic marker, α-SMA, with qRT-PCR did not find significant differences in the corneas of the Group-2 (naïve cohort) and Group-4 (safety cohort) (Fig 3). The comparisons of mMs scores of the wounded eyes with eyedrop (Group-3: therapy cohort) versus without eyedrop (Group-1: injury cohort) exhibited significantly lower mMs scores on day-14 (*p* = 0.0041) and day-28 (*p* = 0.0002) (Fig 1). The IOP and CCT analysis of these two groups are shown in Fig 5A and 5B, respectively.

### Corneal morphology

**Fig 4:**
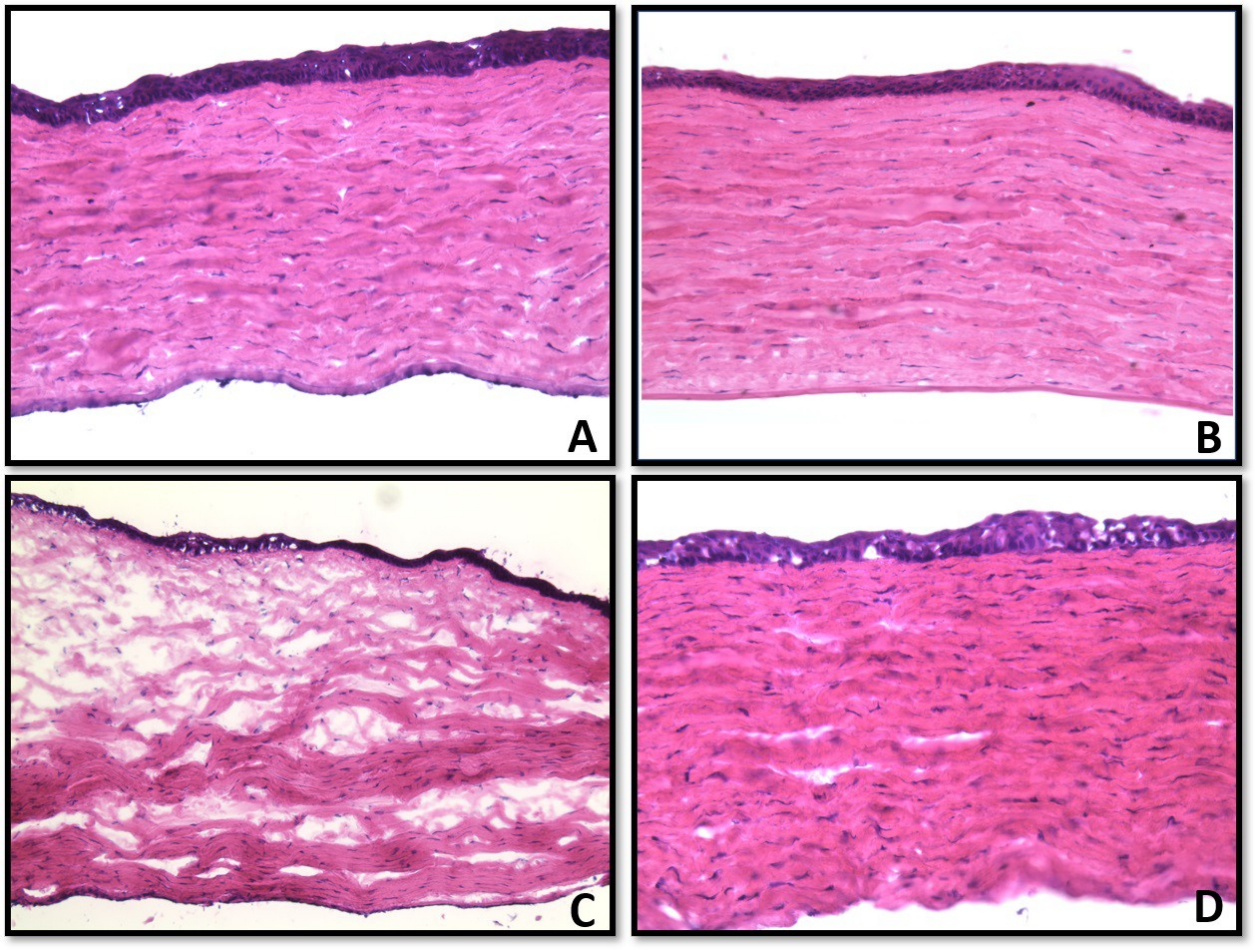
Representative H&E stained corneal tissue sections showing toxicity and efficacy *in vivo*. Corneal sections of naive Group-2 (A), unwounded eyedrop-treated Group-4 (B), alkali wounded Group-1 (C), and alkali wounded and eyedrop-treated Group-4 (D) eyes. Group-2 (A) and Group-4 corneas showed normal corneal morphology. Alkali injury led to significant stromal edema and disorganized collagen, as well as a thin keratinized epithelial layer (C) and eyedrop treatment markedly improved corneal pathology through better collagen fibrils organization, less stromal edema, and re-epithelialization with a full-thickness epithelium (D). Magnification 20x.

H&E staining of unwounded eyes of Group-2 (naïve cohort) and Group-4 (safety cohort) showed no morphological differences in corneal tissues (Fig 4A and 4B). Conversely, H&E stained corneal tissues of wounded eyes of Group-1 (injury cohort) that had no eyedrop demonstrated re-epithelialization with keratinization of the epithelial layer, as well as stromal edema and disorganization of the collagen layers within the stroma (Fig 4C). By contrast, H&E stained wounded corneal tissues of Group-3 (therapy cohort) that received eyedrop showed return of organized stromal collagen fibrils with minimal to no corneal edema, as well as return to normal full thickness epithelium (Fig 4D).

### *In vivo* efficacy

**Fig 5:**
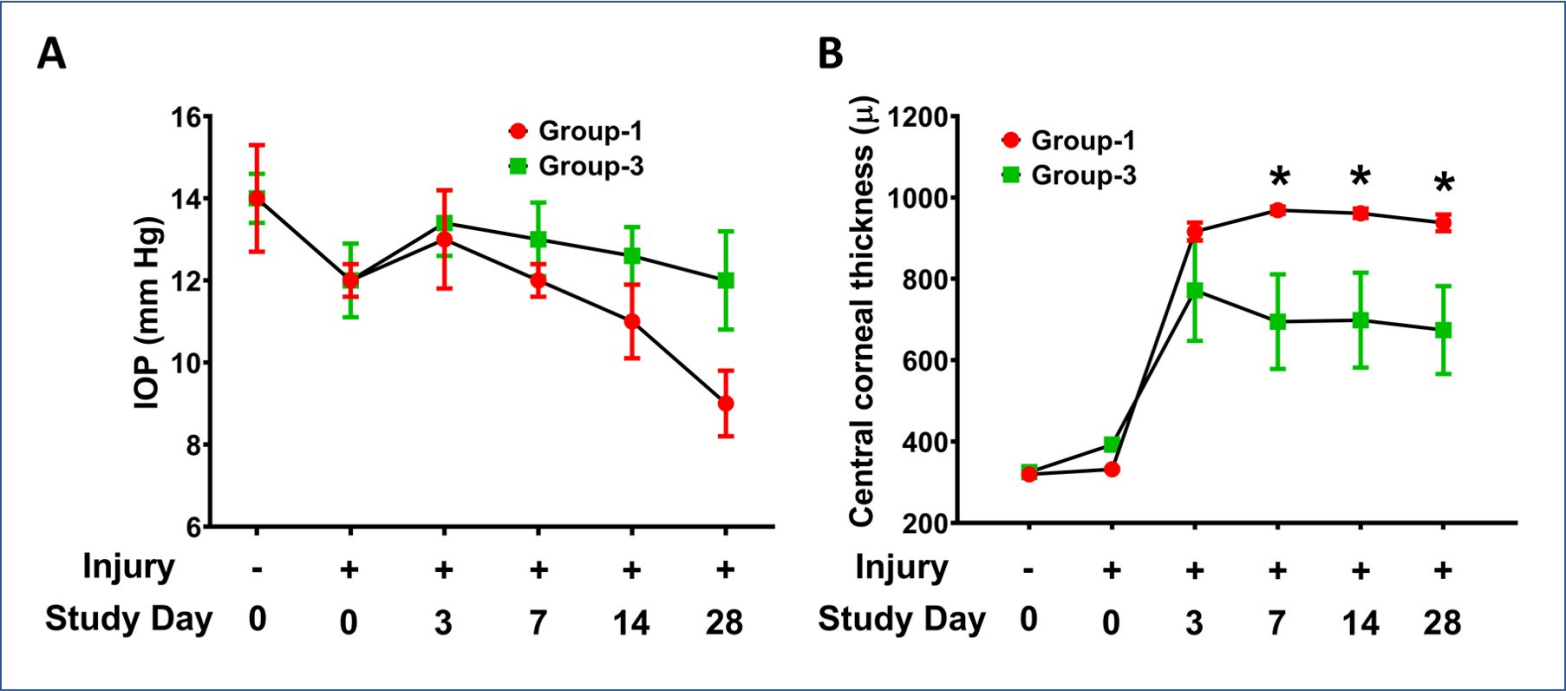
Intraocular pressure and central corneal thickness in wounded eyes -/+ eyedrop. No significant differences in intraocular pressure observed in Group-1 wounded and Group-3 wounded and eyedrop-treated eyes (A). Central corneal thickness was significantly lower in Group-3 wounded and eyedrop-treated eyes at days 7, 14, and 28 (B). (n = 6 for each group; * = *p* value <0.05).

Corneal wounding and ocular health evaluations were performed to evaluate the efficacy of the eyedrop. All employed corneas were healthy prior to the initiation of the study. Following wounding, all corneas developed significant opacity consistent with severe edema and inflammation, and were fluorescein stain positive (data not shown). Corneal wounds had epithelialized in all rabbits by day 5 of the study without complication from infection or self-trauma based on negative fluorescein staining and slit-lamp clinical examination. Likewise, IOP between Group-1 (injury cohort) and Group-3 (therapy cohort) eyes was not significantly different during the study (Fig 5A). Comparison of CCT in Groups 1 and 3 was also performed. The eyedrop treated Group-3 eyes showed significantly decreased CCT at days 7, 14, and 28 (*p*<0.05) than the non-eyedrop treated Group-1 eyes (Fig 5B).

### *In vivo* reduction of fibrosis

**Fig 6:**
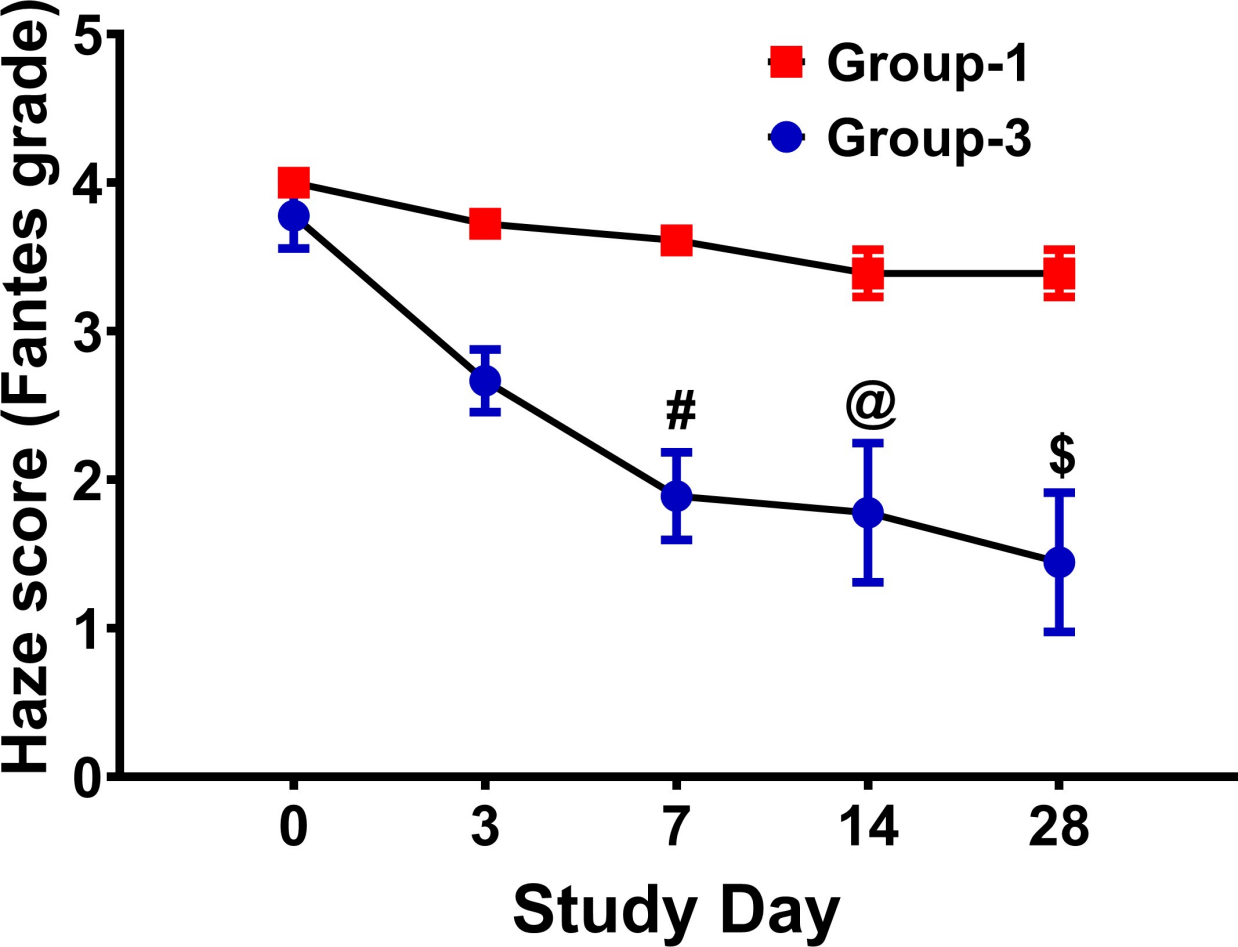
Fantes haze scores in wounded eyes -/+ eyedrop. A significantly lower Fantes scores was noted in Group-3 wounded and eyedrop-treated eyes at day 7, 14, and 28. (n = 6 for each group; @ = *p* value <0.01; # = *p* value <0.001; $ = *p* value <0.0001).

**Fig 7:**
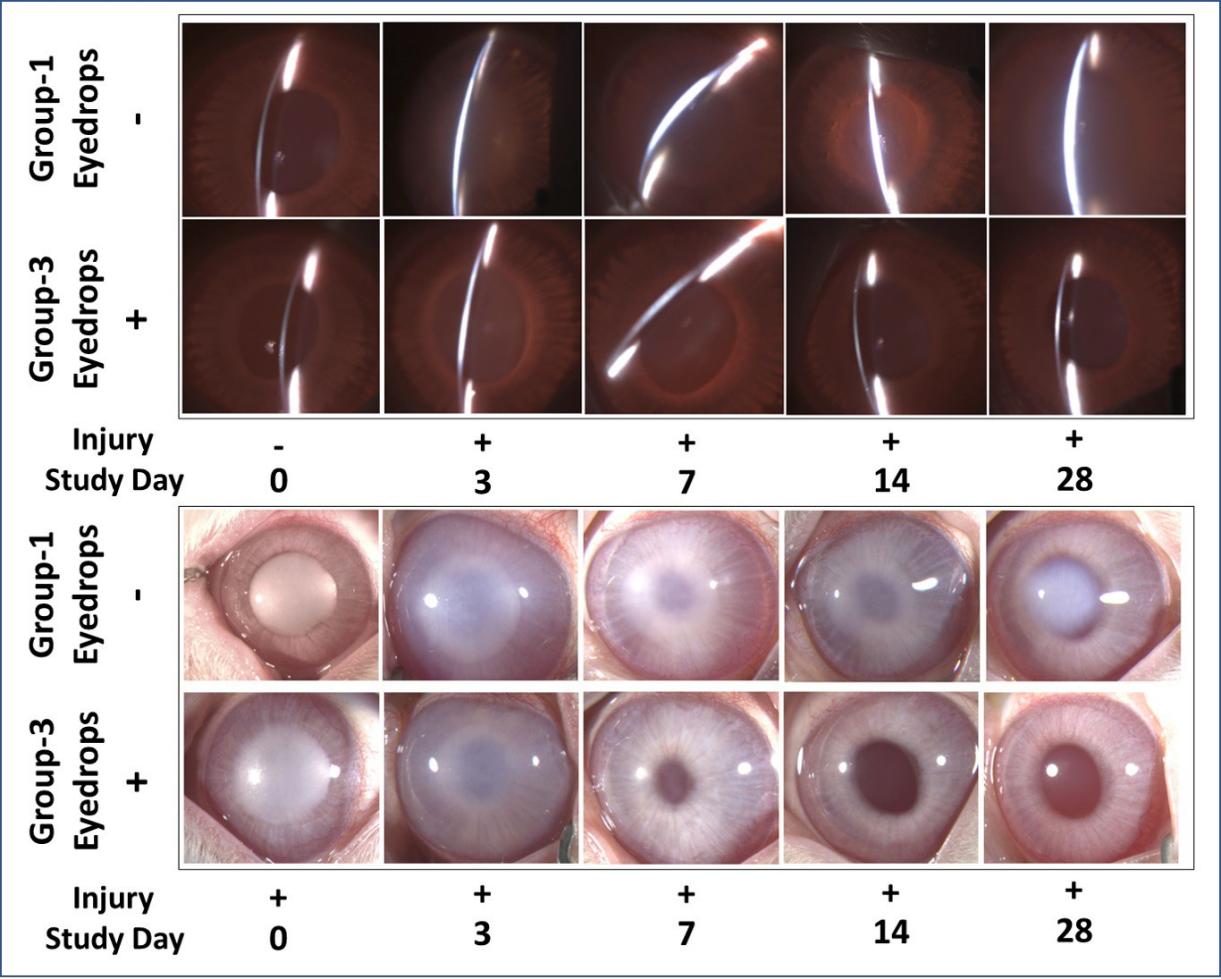
Clinical status of corneal haze in wounded eyes -/+ eyedrop. Representative slit lamp images (A) and stereo biomicroscopy images (B) demonstrating levels of corneal haze from day 0 to 28 in Group-1 and Group-3 eyes. Eyedrop-treated eyes showed substantially reduced corneal haze.

Rabbit eyes that received eyedrop after injury (Group 3: therapy cohort) demonstrated a markedly increased transparency based on significant differences in Fantes scores compared to untreated injured corneas (Group 1: injury cohort) at days—7 (*p* = 0.001), 14 (*p* = 0.0027), and 28 (*p* = 0.0001) (Fig 6). Slit-lamp (Fig 7A) and stereomicroscopic images (Fig 7B) revealed marked differences in corneal haze between injured eyes receiving eyedrop (Group-3: therapy cohort) and the untreated injured eyes (Group-1: injury cohort).

### Profibrotic gene expression and immunohistochemistry

**Fig 8:**
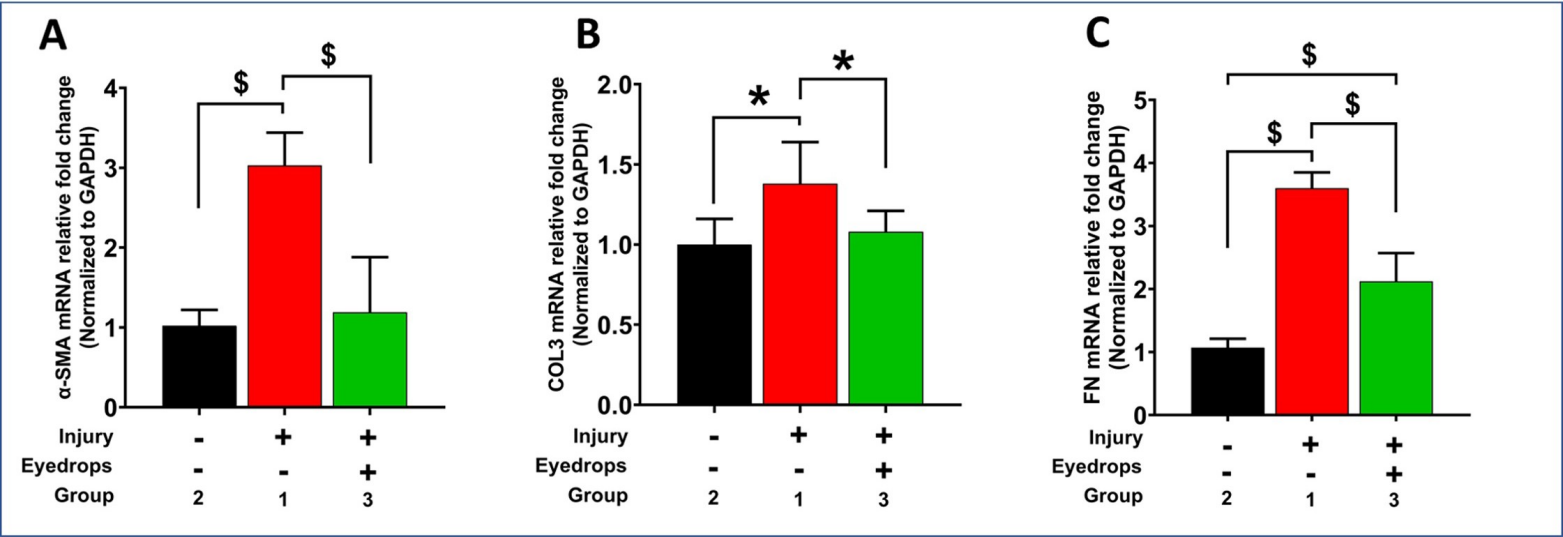
Pro-fibrotic gene expression in naïve and wounded eyes -/+ eyedrop. qRT-PCR analysis showing gene expression of α-SMA (A), Collagen-III (B) and Fibronectin (C) in Group-1 alkali treated, Group-2 naïve, and Group-3 alkali and eyedrop-treated eyes. A significantly increased α-SMA, Collagen III, and Fibronectin expression was detected in alkali treated corneas compared to the naïve corneas, and eyedrop treatment after alkali injury significantly decreased these profibrotic gene’s expression (n = 6 for each group; * = *p* value <0.05; $ = *p* value <0.0001).

**Fig 9:**
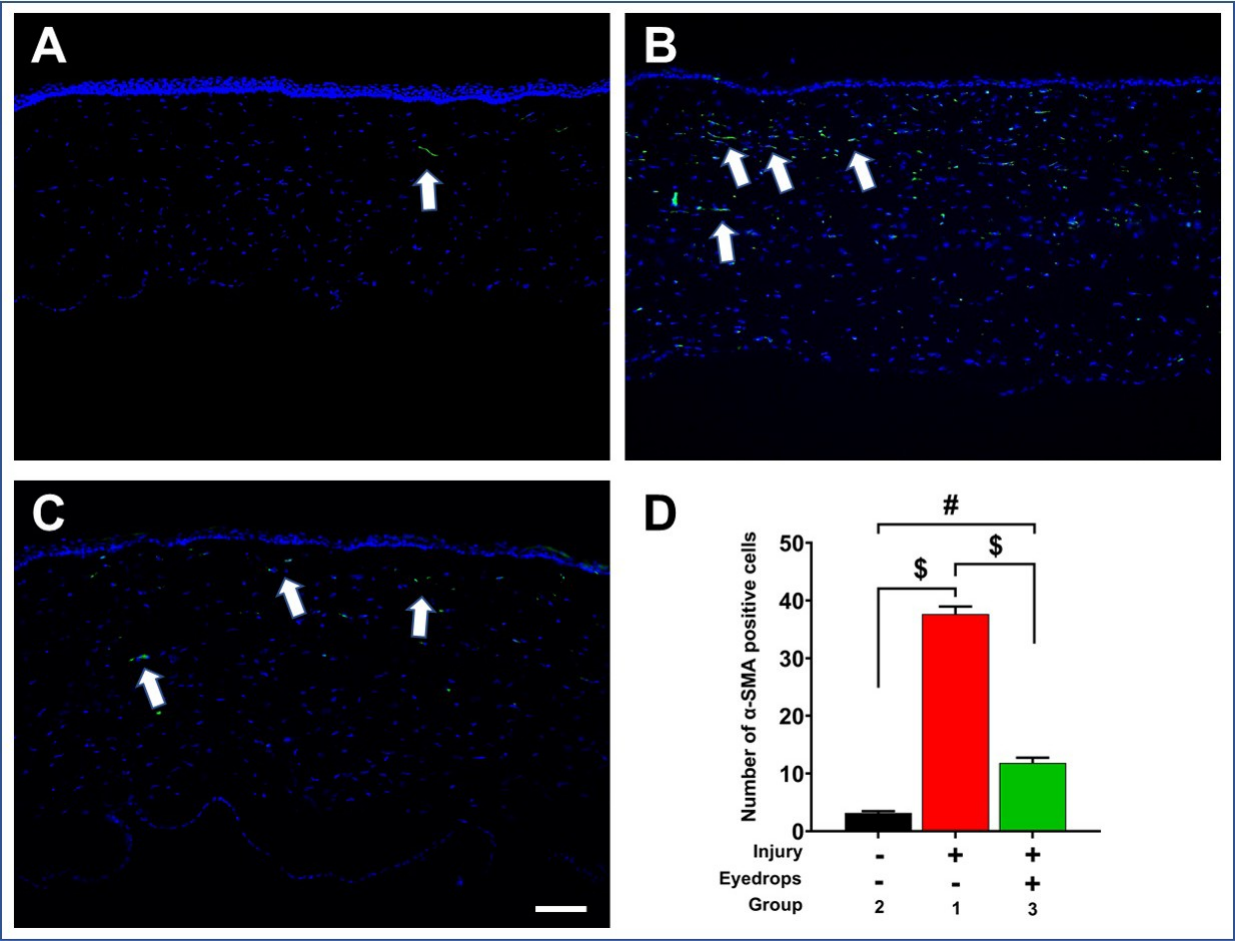
Representative immunofluorescence of α-SMA protein in naïve and wounded eyes -/+ eyedrop. A significantly increased α-SMA+ cells were found in alkali wounded rabbit corneas (B) compared to the naïve corneas (A). Eyedrop treatment after injury significantly decreased α-SMA+ cells in rabbit corneas (C) compared to alkali injured rabbit corneas without eyedrop treatment (B). Panel D shows quantification of SMA+ cells in corneas of 3 groups (n = 6 for each group; # = p<0.001; $ = p<0.0001;). Scale bar = 50μm.

Alkali wounded eyes (Group-1: injury cohort) demonstrated significant upregulation of profibrotic markers compared to naïve corneas (Group-2: naïve cohort) via qRT-PCR, including α-SMA (*p*<0.0001), collagen-3 (p = 0.011), and fibronectin (p<0.0001) as shown in Fig 8. The corneas of wounded rabbit eyes that received eyedrop (Group-3) showed significantly decreased (p<0.0001) expression of α-SMA at day 28 compared to the non-treated corneas (Group-1) (Fig 8A). A similar trend was observed for other tested profibrotic markers collagen-3 (Group-3 vs Group-1, p = 0.0482; Fig 8B) and fibronectin (Group-3 vs Group-1, p<0.0001; Fig 8C). To test if the eyedrop affects expression of profibrotic α-SMA protein, immunofluorescence staining was performed. A significant increase in α-SMA-positive cells (p = <0.0001) in Group-1 wounded corneas (Fig 9B) compared to the Group-2 naïve corneas (Fig 9A) was observed. The eyedrop-treated corneas of Group-3 (Fig 9C) showed significantly reduced α-SMA+ cells compared to untreated injured corneas of Group-1 (p<0.0001). The quantification of α-SMA+ cells in groups 1–3 is shown in Fig 9D.

## Discussion

Corneal wound healing entails a complex cascade of interrelated signaling pathways and cytokines. Formation of corneal haze has been shown to be a result of normal corneal healing pathways which activate and transform fibroblasts to myofibroblasts and induce ingrowth of blood vessels [5, 11, 42, 43]. Our laboratory and others have demonstrated that corneal clarity can be preserved with inhibition of profibrotic and angiogenic factors during the wound healing process, in particular, TGFβ and VEGF [1, 2, 4, 12–14, 17, 21, 24, 32, 40, 44, 45].

Our study has demonstrated that the combination of TRAM-34 and ascorbic acid, when applied topically to wounded rabbit corneas, significantly decreases corneal haze *in vivo*. This was proven by a reduction in clinical parameters including Fantes scores, mMs scores, and CCT. Testing for reduction of fibrotic markers collagen-III, fibronectin, and α-SMA also all demonstrated statistically significant reductions in the presence of these markers. The treatment was well tolerated in unwounded control eyes with no evidence of deleterious clinical side effects based on mMs scoring, CCT, and IOP. Twice daily dosing was used in this study to good effect but it is possible that treatment intervals in higher mammals (dogs, cats, horses, people) may require more frequent application due to differences in tear film and blink rates.

TRAM-34 was developed as a possible therapeutic alternative to agents such as clotrimazole for treatment of ion channel activity disorders including sickle cell disease, and to avoid the systemic toxicity associated with clotrimazole’s inhibition of cytochrome P450 enzymes [22, 46–48]. Since its development, it has been utilized primarily as a research tool in the study of the intermediate conductance Ca^2+^-activated K^+^ channels in various organs including lung, liver, and kidney as well as in certain tumors [23, 26–28, 46, 47, 49, 50]. More recent studies have demonstrated the role of K_Ca_3.1 in the development of fibrosis via activation of TGFβ [27, 50, 51]. Several *in vitro* and *in vivo* studies have shown that TRAM-34 inhibition of the K_Ca_3.1 channel effectively inhibits TGFβ activation and ultimately reduces fibrosis in the lung, liver, and kidneys [26–28].

In ocular tissues, selective blockade of K_Ca_3.1 via application of TRAM-34 has been shown to downregulate TGFβ-activated pro-fibrotic gene expression in both conjunctiva and cornea, and thus reduce activation and differentiation of fibroblasts to myofibroblasts [24, 25]. Another study demonstrated that application of TRAM-34 to alkali-wounded mouse corneas may be beneficial in prevention of corneal angiogenesis via inhibition of epidermal growth factor (EGF) [29]. One study from our group specifically examined the ocular toxicity of TRAM-34 in primary human conjunctival fibroblast cultures by treating the fibroblasts with TRAM-34 at doses of 0, 1, 5, 10, 25, or 50 μM for up to 7 days to evaluate for cellular toxicity [24]. In the aforementioned study by Anumanthan *et al*, there was a moderate decrease in cellular viability at the 50μM dose based on trypan blue exclusion assay, but 25μM and lower doses were well tolerated in the conjunctival cells. Our study supports ocular tolerability of this therapy *in vivo* in the rabbit model with twice daily dosing.

Ascorbic acid has been evaluated for use topically in rabbits at various concentrations ranging from 10mg/mL to 0.5mg/mL after induction of corneal neovascularization via surgical placement of a stromal suture [32]. Lee et al showed significantly reduced presence of markers of angiogenesis VEGF and MMP9 in treated groups compared to controls, as well as a lower ratio of corneal surface area of neovascularization in the treatment groups at 10mg/mL ascorbic acid [32]. Additional data from a recent study using an *in vivo* mouse model of corneal epithelial scraping, with subsequent application of a single dose of topical 10% ascorbic acid, showed a significant improvement in corneal re-epithelialization in the treatment group [31].

A novelty of this study is preparation and evaluation of eyedrop consisting of water soluble vitamin, ascorbic acid, and a highly selective and potent inhibitor of the intermediate-conductance Ca2+-activated K+ channel (K_Ca_3.1), TRAM-34, that does not block cytochrome P450. Also, previous studies of topical TRAM-34 found it highly effective in preventing fibrosis in ocular and non-ocular systems *in vitro* and *in vivo* [24, 25, 29]. Likewise, ascorbic acid has been previously evaluated in rabbits *in vivo* and shown great success in treating corneal ulcers and improving corneal healing [30, 31, 33, 52]. To the best of authors knowledge, a combination of these agents has never been tested previously. This is the first study formulating, preparing, and evaluating the safety and efficacy of a bimodal eyedrop consisting of TRAM-34 and ascorbic acid in rabbits *in vivo*. Another strength of the study is use of alkali dosing in rabbit that produces fibrosis in the cornea without significant neovascularization.

There are certain limitations to this study. For example, no direct comparisons of the anti-fibrotic effect of combination eyedrop with TRAM-34 or ascorbic acid alone were performed, the changes in cellular and molecular parameters in corneal tissues were evaluated only at one time point (28 days), and minimal efforts were made to characterize underlying mechanisms. Also, we did not evaluate effects of TRAM-34 on corneal epithelial and stromal fibroblast cells despite the fact that potassium channels could modulate cellular proliferation, an important factor in corneal wound healing. Our future studies will address these limitations.

We observed downregulation of multiple fibrotic markers associated with TGFβ-mediated fibrosis, including collagen III, fibronectin, and α-SMA, in this study’s therapy cohort (group 3). The reduction of these markers supports our conclusion that our therapy targets TGFβ and has antifibrotic properties in rabbit corneas. In this study, anti-angiogenic markers such as VEGF and MMP9 were not specifically tested, as that was not the primary aim of the current research. However, future studies may examine other markers to help further delineate the mechanisms of both TRAM-34 and ascorbic acid and their combined effect on corneal stromal wound healing.

The combination of TRAM-34 and ascorbic acid applied topically was well tolerated and effective in prevention of corneal fibrosis through inhibition of TGFβ-mediated fibroblast migration and myofibroblast differentiation. Further studies are needed to determine the safety and efficacy in other species as well as the optimal dosing regimen. Additionally, further study is required to examine the efficacy of this therapy in treating an established fibrotic corneal lesion.
